# Influence of the Self-Perception of Old Age on the Effect of a Healthy Aging Program

**DOI:** 10.3390/jcm7050106

**Published:** 2018-05-07

**Authors:** Víctor Manuel Mendoza-Núñez, Elia Sarmiento-Salmorán, Regulo Marín-Cortés, María de la Luz Martínez-Maldonado, Mirna Ruiz-Ramos

**Affiliations:** 1 Unidad de Investigación en Gerontología, Facultad de Estudios Superiores Zaragoza, Universidad Nacional Autónoma de México (UNAM), Guelatao N° 66, Col. Ejército de Oriente, 09230 Ciudad de México, Mexico; elios_yat@hotmail.com (E.S.-S.); marilumtz05@yahoo.com.mx (M.d.l.L.M.-M.); mirna1411@yahoo.com.mx (M.R.-R.); 2 Escuela Nacional de Trabajo Social, Universidad Nacional Autónoma de México, CP 04510 Ciudad de México, Mexico; menuvi05@yahoo.com.mx

**Keywords:** self-perception of old age, self-care, healthy aging, Mexican community-dwelling older people

## Abstract

It has been shown that health programs are useful for the prevention and control of chronic diseases in community-dwelling older people; however, a negative self-perception of old age could have an effect on the results. Therefore, our aim was to evaluate the effect of a healthy aging program linked to self-perception of old age in Mexican community-dwelling older people. A pre-test/post-test single-group design study was conducted in a convenience sample of 64 older people who undertook the entire healthy aging program workshop (five months’ duration). We measured self-perception of old age, efficacy of self-care, blood glucose concentration, anthropometric measures, and blood pressure before and after the workshop. A statistically significant decrease in blood glucose concentration was observed (baseline 136 ± 50 vs. post-intervention, 124 ± 45 ± 29 mg/dL, *p* < 0.01), LDL (baseline 153 ± 47 vs. post-intervention, 130 ± 36 mg/dL, *p* < 0.01), systolic blood pressure (130 ± 20 vs. 119 ± 11 mm/Hg, *p* < 0.001), and diastolic blood pressure (75 ± 9 vs. 72 ± 7 mm/Hg, *p* < 0.05) after community intervention. However, when we analyzed the data regarding self-perception, we found that this difference was only maintained in the subgroup of older adults with a positive self-perception of old age. Our findings suggest that the self-perception of old age influences the effect of healthy aging programs on the health of community-dwelling older people.

## 1. Introduction

The aging population represents major challenges for all societies, with profound consequences for individual and community life, as well as repercussions in the various spheres of human existence, including social, economic, political, cultural, and health [1] sectors. In Mexico, 10.4% of the total population (12.4 million) is considered to be old (≥60 years); the average life expectancy at the age of 60 is 22 years [2]. This situation has legal, social, economic, and above all, health implications. Additionally, there is high prevalence of chronic degenerative diseases linked to disability and poverty, which has an impact on the quality of life of the elderly and their families [3]. For this reason, it is necessary to propose and develop programs for promoting successful aging in the community, which are based on scientific knowledge and are adapted to the socio-cultural context of each country.

Our working group has developed a model of community gerontology for ensuring active and healthy aging, which considers self-care, mutual help, and self-promotion as key strategies. These are achieved by establishing the adoption and maintenance of healthy lifestyles [4]. In this sense, the aim of this model is to provide older people with the basic knowledge of gerontology for self-care, mutual help, and self-promotion, related to preventing and controlling chronic diseases that have high prevalence among aged communities. As a result, individuals can better maintain their functionality and well-being [5]. We have observed the positive effect of implementing this model on the health of older people [6]. Likewise, we have observed changes in the negative self-perception of aging in older people from four countries, who underwent training in a university program for older adults as part of a multicentric study [7].

It has also been demonstrated that self-care and health are negatively impacted by negative self-perceptions on aging held by the elderly themselves. In contrast, a positive self-perception of aging has a beneficial effect on health, functionality, and longevity [8,9,10].

In this context, we hypothesized that there could be a relationship between the self-perception of old age and the effectiveness of implementing healthy aging programs in the community. Therefore, the aim of this study was to evaluate the effect of a healthy aging program, and the impact of self-perception of old age in Mexican community-dwelling older people.

## 2. Material and Methods

### 2.1. Study Design and Subjects

**Figure 1:**
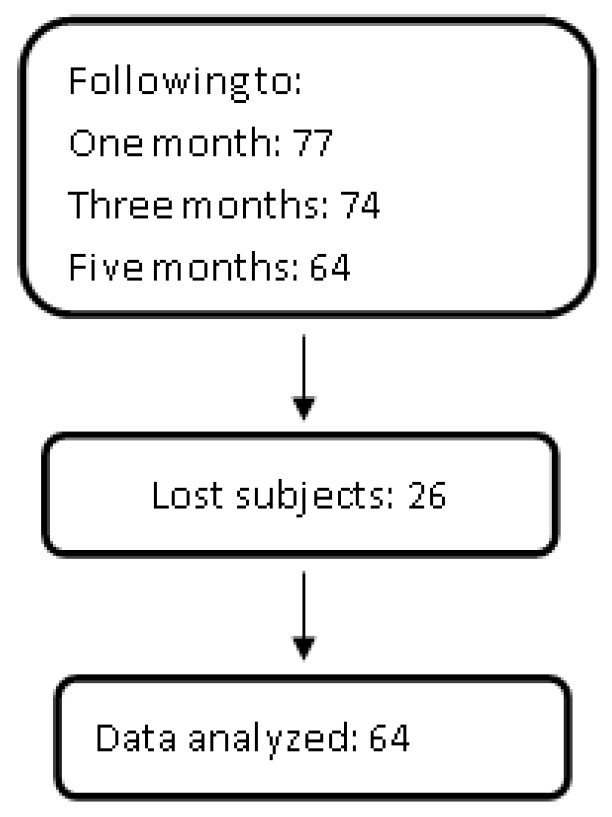
Outline of the study.

Informative brochures were distributed in the community specifying the objectives of the study and admission criteria: (i) interest in participating in the study; (ii) literacy, low level of schooling (≤6 years of education), high level of schooling (≥7 years of education); and (iii) an absence of handicapping illnesses or serious visual or auditory disabilities. A pre-test/post-test single-group design study was conducted in a convenience sample of 90 community-dwelling older people living in Hidalgo State, Mexico (69 women and 21 men). A total of 13 subjects left the program during the first month, due to the fact that they no longer had enough time to attend the workshop (they took care of grandchildren and/or did housework); and 3 other subjects left the program in the third month due to illness. Therefore, 64 (52 women and 12 men) participants completed the “healthy aging program” workshop. The subjects agreed to participate in the study after providing informed consent. The Ethics Committee of the Universidad Nacional Autónoma de México (UNAM) Zaragoza Campus approved the research protocol for this study (PAPIME PE305516). We measured the self-perception of old age, efficacy of self-care, anthropometry and blood pressure, and biochemical parameters before and after five months of intervention (Figure 1).

### 2.2. Assessment of Self-Perception of Old Age

“Self-rated attitudes towards old age” is an instrument that was developed by the University of the State of Mexico and was adapted by experts in the field of gerontology (Table 1) [11]. The reliability of the instrument is α = 0.83.

Percentiles were calculated by setting cut-off points at the 25th and 75th percentile score for positive and negative self-perception, respectively. The population located in the first quartile (Q_1_) was classified as having positive self-perception (score ≤ 44), while those in the fourth quartile (Q_4_) was classified as having negative self-perception (score ≥ 59).

### 2.3. Assessment of Anthropometric Measurements and Blood Pressure

After a clinical history was obtained and a physical examination was performed, we obtained the following anthropometric measurements. Weight was measured while the subject was wearing underwear and a clinical smock and was in a fasted state (after bowel evacuation). A Torino scale (Tecno Lógica, Mexicana, México, TLM) was used and calibrated before each weight measurement. Height was obtained with an aluminum cursor stadiometer graduated in millimeters. The subject was barefoot with their back and head in contact with the stadiometer in the Frankfurt horizontal plane. Body mass index (BMI) was calculated by dividing the weight (in kilograms) by the squared value of height (in m^2^). Waist circumference was measured in centimeters with a tape measure at the level of the umbilicus to the nearest 0.5 cm. Blood pressure was measured in both arms three times in the morning and in a fasting condition, or 2 h after breakfast in both sitting and standing positions. A mercurial manometer was used to measure the blood pressure. Subjects with pseudohypertension were identified by applying the Osler technique, which involves feeling the radial pulse when the manometer registers values above the true systolic pressure. Blood pressure was taken by medical technicians, who had attended training sessions to standardize the procedures. The technicians were supervised to avoid possible biases in the measurements.

### 2.4. Assessment of Biochemical Parameters

Serum levels of glucose, cholesterol, triglyceride and HDL were determined using an Autoanalyzer Vitalab Eclipse from Merck (Dieren, The Netherlands). Specifically, glucose levels were measured using the glucose oxidase method (normal range, 63–120 mg/dL). Cholesterol was analyzed using the cholesterol oxidase phenol 4-aminoantipyrine peroxidase (CHOD-PAP) technique (normal range, 168–200 mg/dL), and triglycerides by the glycerol phosphate oxidase-Trinder (GPO-Trinder) technique (normal range, 89–190 mg/dL), while HDL was assessed using the same technique used to analyze cholesterol after precipitation of low-density and very low-density lipoproteins using a phosphotungstic acid–magnesium chloride solution (normal range, 42–77 mg/dL).

### 2.5. “Healthy Aging Program” Workshop

A program was designed consisting of a 100-h workshop that integrated both theoretical and practical components (20 sessions of 5 h per week) [4]. An introductory textbook entitled “Community Gerontology” was designed specifically for this project, which covered selected topics relevant to the type of activities and responsibilities expected from a gerontological promoter [12]. A complementary workbook was designed including quizzes on all revised topics. The topics of the workshop were selected and approved by a panel of four gerontologists based on their knowledge of community gerontology regarding age-related changes in the biological, psychological and social area, as well as in the prevention of chronic diseases, healthy lifestyles during the aging process, empowerment, social support networks and ageism (Table 2). The workshop was conducted by four trained gerontologists.

### 2.6. Statistical Methods

Descriptive statistics were expressed as the mean and standard deviation or in percentages. Student’s *t*-test was used to compare groups. Spearman’s correlations were used to assess correlations between self-perception of aging and related self-efficacy of self-care, anthropometric measures and biochemical parameters. *p*-values < 0.05 were considered to be significant. Statistical analysis was performed using SPSS version 20.

## 3. Results

**Figure 2:**
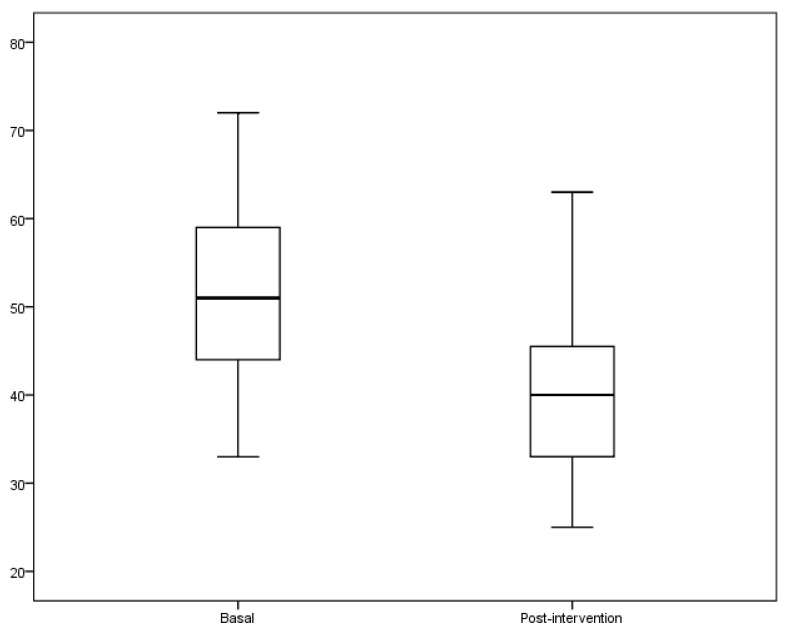
Mean ± SD score on self-rated negative attitudes to old age basal and post intervention. The graph shows the results of the community intervention in the score with “health promotion and self-care in the old age” workshop. The score shows a decrease statistically significant after intervention (baseline, 51 ± 10 vs. post-intervention, 40 ± 9, *p* < 0.001). Student’s *t* test, the values expressed as the mean ± standard deviation.

Table 3 shows the socio-demographic characteristics and health status of the study population. In the assessment of self-perception of aging, there was a significant decrease in the mean score of the negative attitudes on the aging scale in the study population after the intervention (baseline of 51 ± 10 vs. post-intervention of 40 ± 9, *p* < 0.01) (Figure 2), and therefore, an enhancement in positive self-perception of aging. Likewise, in the analysis by quartiles (Q) of the self-perception of old age after the workshop, a statistical decrease was observed, with significant changes in the scores of the Q_1_ (baseline of 51 ± 10 vs. post-intervention of 40 ± 9, *p* < 0.05), Q_3_ (baseline of 55 ± 1 vs. post-intervention of 40 ± 9, *p* < 0.05) and Q_4_ (baseline of 63 ± 4 vs. post-intervention of 46 ± 10, *p* < 0.05) (Table 4).

Regarding to the influence of education on self-perception of old age, before the training a significantly higher score was observed in the low schooling group (LS, ≤6 school years) compared to high schooling group (HS, ≥7 school years) (55.2 ± 9.7 vs. 46.3 ± 1, *p* < 0.001). Likewise, in both groups, a statistically significant decrease in the score was observed, being more evident in the low schooling group (LS: baseline 55.2 ± 9.7 vs. post-intervention 41.8 ± 10, *p* < 0.001, HS: baseline 46.3 ±1 vs. post-intervention 38 ± 6.6, *p* < 0.05).

A significant decrease in blood glucose concentration was found in the total population (baseline of 137 ± 50 vs. post-intervention of 124 ± 45, *p* < 0.01), although this decrease was only maintained in the positive self-perception group (baseline of 134 ± 47 vs. post-intervention of 114 ± 29 mg/dL, *p* < 0.05). On the other hand, no significant changes were observed in blood cholesterol concentration in the analysis of the total population (baseline of 224 ± 42 vs. post-intervention of 218 ± 44 mmol/dL, *p* > 0.05). However, when we analyzed the data according to self-perception groups, only the group with positive self-perception showed a statistically significant decrease (baseline of 223 ± 37 vs. post-intervention of 202 ± 41 mmol/dL, *p* < 0.05) as the group with negative self-perception did not show statistically significant differences. Likewise, a statistically significant decrease in blood LDL concentration was found in the total population (baseline of 153 ± 48 vs. post-intervention of 130 ± 36, *p* < 0.01), although this was only maintained in the positive self-perception group (baseline of 159 ± 40 vs. post-intervention of 116 ± 32, *p* < 0.05). Regarding the systolic blood pressure, a statistically significant decrease in the total population was observed without differences between the groups with positive and negative self-perception (Table 5).

Evaluation of the self-efficacy of self-care assessment revealed significantly higher health care and sleep hygiene scores in the total population after the intervention (Table 6). However, when we analyzed the data according to self-perception groups, only the group with positive self-perception showed significantly higher health care and self-esteem scores, while there was no significant change in the group with a negative self-perception of aging (Table 7). On the other hand, regarding to the influence of education on self-efficacy of self-care actions, no statistically significant change was found in the score to health care in the LS group (baseline 7.6 ± 1.3 vs. post-intervention 7.8 ± 1.3, *p* > 0.05), in contrast to the HS group, which showed a significantly higher score (baseline 7.8 ± 1.3 vs. post-intervention 8.7 ± 0.8, *p* < 0.01). However, we did not observe statistically significant differences in the other healthy lifestyles when were compared the low and high schooling groups.

Additionally, a negative self-perception of ageing score correlated with higher serum concentrations of triglycerides (*r* = 0.22, *p* < 0.05), cholesterol (*r* = 0.31, *p* < 0.01), low-density lipoprotein (*r* = 0.31, *p* < 0.01) and glucose (*r* = 0.29, *p* < 0.01).

## 4. Discussion

It has been shown that health promotion and education programs are useful for the preventive control and management disease in community-dwelling older people [13,14,15]. In this sense, it has been demonstrated that education provided to the elderly regarding the biological, psychological and social changes related to aging and self-care improves self-perception of aging and group stereotypes, and significantly reduces any negative effects by improving emotional balance [7]. Thus, we observed an improvement in the positive perception of old age following implementation of the “healthy aging” workshop in our study. These findings support the theory that objective knowledge about biological, psychological and social changes related to aging improves the self-perception of old age in the elderly [7,16].

It has been pointed out that the education of the older adults influences the self-perception of old age, observing that older adults with higher schooling have better the perception of old age compared to the negative self-perception of the elderly with low schooling [17,18]. In this sense, in our study in the measurement of the self-perception of the old age before the workshop, we observed a better perception of the old age in the group with higher schooling in comparison with the low schooling group, although after the workshop both groups showed better perception of old age. These findings are in agreement with other studies [19,20], although it has also been found that education does not influence the self-perception of aging [21], which suggests that, besides the years of schooling, social representations of old age are a determining factor [22].

Additionally, after the intervention, the study population showed positive changes in anthropometric variables, such as BMI, and clinical and biochemical parameters, such as systolic and diastolic blood pressure, as well as serum levels of glucose and LDL. This suggests that the study participants incorporated the knowledge acquired during the “healthy aging” workshop into self-care. Similarly, the serum levels of glucose, cholesterol, low-density lipoprotein and systolic blood pressure decreased following the intervention in participants with positive self-perception of aging, while there was only a reduction in systolic blood pressure in the group with negative self-perception of aging. These findings eliminate the confounding factor of the overall score of the population by allowing a more precise assessment of the effects of positive and negative self-perceptions of aging.

These results support the assertion made by Allen [23] that “recurrent experiences with negative stereotypes combined with discrimination may make ageism a chronic stressor in the lives of older adults” and therefore, “may increase the risk of chronic disease, mortality, and other adverse health outcomes”.

Evaluation of the self-efficacy scores after the intervention revealed a significant improvement in health care and self-esteem in the group with positive self-perception of aging, while the group with negative self-perception of aging showed no changes. These findings support the benefit of health promotion in changing attitudes held by the elderly towards old age and health self-care and self-recognition [8,9,24].

In this context, Fernández-Ballesteros observed that possessing positive self-perception of aging moderates the threat posed by stereotypes, and that positive information promotes better memory performance in older adults [25].

Another important finding observed in our study was the relationship between positive self-perception and the improved scores of self-efficacy of care for health, self-esteem, nutrition, sleep hygiene and health. In contrast, negative self-perceptions of aging after intervention correlated with higher serum concentrations of triglycerides, cholesterol, low-density lipoprotein and glucose.

The study demonstrated the importance of imparting knowledge to the elderly regarding the biological, psychological and social changes related to aging and old age, combined with self-care strategies related to self-perceptions of aging and its effects on health.

Prejudices against the elderly and stereotypes of old age and aging go back a long way in human history, and have had both positive and negative components. Positive perceptions of aging were espoused by Plato and Cicero, while Aristotle and Seneca likened old age to an incurable disease [16]. The “Terror Management Theory” explains ageism as an unconscious defensive strategy employed by younger adults against their anxiety surrounding death. As for the elderly, their negative self-perceptions have a social origin that is part of the “Social Identity Theory” [26].

The negative view of aging has led to numerous scientific “anti-aging” strategies designed to “reverse aging”, such as the use of pills [27]. These efforts strengthen the negative social perception of aging and old age. In response, Palmore noted the need to develop “anti-ageism” strategies [28].

### Limitations

This is a pilot study and the sample size was not representative. Thus, it is necessary to conduct a quasi-experimental study with a control group and a representative sample to confirm our findings. The effect related to socialization influence was not controlled. The data on behavioral changes of self-care to health before and after the workshop were self-reported by the participants, so we could not be certain of the reported changes.

## 5. Conclusions

Our findings suggest that educating older adults through “health promotion and aging” has a positive effect on encouraging positive self-perception of aging, self-care and health status. The effectiveness of the implementation of self-care models for older adults at the community level of our country has recently been reported by other research groups [29,30]. For this reason, it would be desirable for state public health programs to consider developing self-care models as a priority for achieving healthy aging with consideration of the positive self-perception of aging.

## Figures and Tables

**Table 1:** Self-perception scale of old age.

No.	Item
1	It makes me sad to be elderly.
2	It is unpleasant to have wrinkles.
3	Old age scares me.
4	I do not like having graying hair.
5	The elderly have bad breath.
6	It is awful losing mental abilities with old age.
7	Older people become fools and repeat themselves.
8	With aging comes sadness and loneliness.
9	Women with graying hair are unattractive.
10	Older people are abandoned.
11	Men with age-related baldness are unattractive.
12	Elderly women should take steps to look younger.
13	Being old is depressing.
14	Older people are smelly.
15	It is easy to fool old people.
16	With aging, independence is lost.
17	The elderly cause many problems.
18	The elderly do not have skills such as driving cars.
19	The elderly are greedy.
20	Nursing homes are depressing.
21	I fear being useless when I am old.

Scores: 1 = completely disagree, 2 = moderately disagree, 3 = moderately agree; 4 = completely agree. A high score is considered to represent a negative self-perception.

**Table 2:** Workshop topics revised in the healthy aging program.

Topics
Concept of aging and old age Age-related biological, psychological and social changes Healthy aging and functionality Mouth and teeth care in the elderly Skin, nails and foots care in the elderly Nutrition in the aging Physical exercise in the aging Prevention of falls in the elderly Sexuality in older adults Self-esteem and aging Ageism Active aging and empowerment Life quality and aging Social support networks Self-care, mutual-help and self-promotion Diabetes mellitus and arterial hypertension Mild cognitive impairment and memory training Depression Elder abuse Thanatology

**Table 3:** Sociodemographic characteristics and health status of the study population.

	Frequencies, *n* = 64 (%)
Age (mean ± SD)	66 ± 3
60 a 69 years	49 (77)
≥70 years	15 (23)
Sex	
Woman	52 (81)
Man	12 (19)
Civil status	
Single	20 (31)
Married	30 (47)
Widower	14 (22)
School years (mean ± SD)	7 ± 5
Literacy	3 (4)
1–6 years	35 (55)
7–9 years	11 (18)
≥10 years	15 (23)
Diagnosis	23 (36)
Healthy	9 (15)
DM	13 (29)
HBP	19 (30)
DM + HBP	

Data are frequencies and percentages. DM, diabetes mellitus type 2; HBP, high blood pressure.

**Table 4:** Mean score of positive and negative self-perception of aging group’s baseline and post-intervention.

Self-Perception of Aging	Baseline	Post-Intervention
Total (*n* = 64)	51 ± 10	40 ± 9 ***
Q_1_ (*n* = 18)	39 ± 3	33 ± 4 ***
Q_2_ (*n* = 17)	48 ± 2	46 ± 5
Q_3_ (*n* = 10)	55 ± 1	40 ± 9 ***
Q_4_ (*n* = 19)	63 ± 4	46 ± 10 ***

Mean score of self-perception of old age in presented by quartiles (Q). Quartile 1 (Q_1_) was located in the 25th percentile score ≤ 44; quartile 2 (Q_2_) was located in the 26th to 50th percentile score 45–51; quartile 3 (Q_3_) was located in the 51th to 75th to score 52–58 was; and quartile 4 (Q_4_) was located in the 76th to 100th percentile score ≥ 59. Data presented are means and standard deviations. Paired *t*-test, *** *p* < 0.0001.

**Table 5:** Effect of healthy aging program linked to positive and negative self-perception old age.

	Total *n* = 64	Q_1_ (*n* = 18) Positive Self-Perception	Q_4_ (*n* = 19) Negative Self-Perception
Glucose (mg/dL)			
Baseline	137 ± 50	134 ± 47	144 ± 46
Post-intervention	124 ± 45 **	114 ± 29 *	137 ± 53
Cholesterol (mmo/dL)			
Baseline	224 ± 42	223 ± 37	234 ± 35
Post-intervention	218 ± 44	202 ± 41 *	229 ± 38
Tryglicerides (mg/dL)			
Baseline	165 ± 92	139 ± 52	160 ± 92
Post-intervention	159 ± 75	132 ± 66	158 ± 73
LDL (mg/dL)			
Baseline	153 ± 48	159 ± 40	164 ± 56
Post-intervention	130 ± 36 **	116 ± 32 *	140 ± 32
HDL (mg/dL)			
Baseline	56 ± 15	53 ± 14	58 ± 14
Post-intervention	58 ± 14	58 ± 13	61 ± 17
Weight (kg)			
Baseline	67 ± 12	66 ± 13	66 ± 10
Post-intervention	66 ± 10	65 ± 11	66 ± 10
BMI (weight/height^2^)			
Baseline	29 ± 4	27 ± 3	30 ± 5
Post-intervention	28 ± 3 *	27 ± 3	29 ± 4
SBP			
Baseline	131 ± 20	124 ± 7	133 ± 15
Post-intervention	119 ± 12 **	115 ± 7 *	124 ± 9 **
DBP			
Baseline	76 ± 9	74 ± 7	74 ± 10
Post-intervention	73 ± 7 *	72 ± 8	75 ± 6

BMI: body mass index; SBP: systolic blood pressure, DBP: diastolic blood pressure. Population considered to show positive self-perception of old age was the included in quartile 1 (Q_1_) that scored ≤44, located in between 1st and 25th percentiles, and negative self-perception of aging in the included in quartile 4 (Q_4_) score ≥ 59 located between the 76th and 100th percentiles. Data presented are means and standard deviations. Paired *t*-test * *p* < 0.05, ** *p* < 0.01.

**Table 6:** Self-efficacy of self-care actions related to healthy lifestyles.

Healthy Lifestyles (*n* = 64)	Baseline	Post-Intervention	*p* Value
Health care	7.7 ± 1.2	8.2 ± 1.2	0.040
Self-esteem	8.3 ± 1.1	8.4 ± 1.5	0.814
Healthy food	7.9 ± 1.3	8.1 ± 1.4	0.216
Physical exercise	8.0 ± 1.6	8.5 ± 1.2	0.21
Sleep hygiene	7.4 ± 2.0	8.2 ± 1.5	0.015
Body hygiene	8.4 ± 1.4	8.9 ± 1.1	0.060
Healthy environmental	8.4 ± 1.4	8.5 ± 1.3	0.585

Data presented are means and standard deviations. Data presented are averages and standard deviations. Paired *t*-test.

**Table 7:** Self-efficacy of self-care actions related to positive and negative self-perception of old age.

	Baseline	Post-Intervention	*p* Value
Positive self-perception of old age (score ≤ 44)			
Health care	8.0 ± 1.0	8.9 ± 0.8	0.004
Self-esteem	8.0 ± 0.8	9.2 ± 1.0	0.008
Healthy food	7.9 ± 1.0	8.5 ± 1.0	0.145
Physical exercise	8.4 ± 1.0	8.4 ± 1.0	1.000
Sleep hygiene	7.7 ± 1.0	8.5 ± 1.0	0.154
Body hygiene	8.9 ± 1.0	9.2 ± 1.0	0.205
Healthy environmental	8.9 ± 1.0	9.0 ± 1.0	0.668
Negative self-perception of old age (score ≥ 59)			
Health care	7.0 ± 1.0	7.0 ± 1.0	1.000
Self-esteem	8.0 ± 1.0	8.0 ± 1.0	1.000
Healthy food	8.0 ± 1.0	7.0 ± 1.0	0.440
Physical exercise	8.0 ± 1.0	8.0 ± 1.0	1.000
Sleep hygiene	6.0 ± 2.0	7.0 ± 1.0	0.538
Body hygiene	8.0 ± 1.0	8.0 ± 1.0	1.000
Healthy environmental	8.0 ± 1.0	8.0 ± 1.0	1.000

Population considered as showing positive self-perception of old age was the included in quartile 1 (Q_1_) that scored ≤44, located in between 1st and 25th percentiles, and negative self-perception of aging in the included in quartile 4 (Q_4_) score ≥59, located between the 76th and 100th percentiles. Data presented are means and standard deviations. Data presented are mean and standard deviation. Paired *t*-test.
